# CRISPR-Cas-Based Engineering of Probiotics

**DOI:** 10.34133/bdr.0017

**Published:** 2023-09-29

**Authors:** Ling Liu, Shimaa Elsayed Helal, Nan Peng

**Affiliations:** ^1^ National Key Laboratory of Agricultural Microbiology, Hubei Hongshan Laboratory, College of Life Science and Technology, Huazhong Agricultural University, Wuhan 430070, Hubei, China.; ^2^ CABIO Biotech (Wuhan) Co. Ltd., Wuhan, China.

## Abstract

Probiotics are the treasure of the microbiology fields. They have been widely used in the food industry, clinical treatment, and other fields. The equivocal health-promoting effects and the unknown action mechanism were the largest obstacles for further probiotic’s developed applications. In recent years, various genome editing techniques have been developed and applied to explore the mechanisms and functional modifications of probiotics. As important genome editing tools, CRISPR-Cas systems that have opened new improvements in genome editing dedicated to probiotics. The high efficiency, flexibility, and specificity are the advantages of using CRISPR-Cas systems. Here, we summarize the classification and distribution of CRISPR-Cas systems in probiotics, as well as the editing tools developed on the basis of them. Then, we discuss the genome editing of probiotics based on CRISPR-Cas systems and the applications of the engineered probiotics through CRISPR-Cas systems. Finally, we proposed a design route for CRISPR systems that related to the genetically engineered probiotics.

## Introduction

Probiotics are defined as “live microorganisms” that confer health benefits on the host when administered in appropriate amounts [1]. Common probiotics include *Lactobacillus*, *Bifidobacterium*, and *Saccharomyces*, etc., although probiotics are not, conceptually and by definition, phylogenetic units [2]. Traditionally, probiotics have been mainly explored as an important raw material for the production of fermented foods. Later, researchers started to pay great attention to some metabolites of probiotics that contribute to anti-inflammatory activity [3] or even brain function [4], promoting the application of probiotics in some new fields such as therapeutic industry and animal health. For example, as the products of most probiotics, short-chain fatty acids provide much energy for colonic epithelial cells to contribute to the integrity of the gut structure, as well as reduce local pH values to inhibit the growth of pathogenic bacteria [5,6]. Research on the interactions between bioactive metabolites and host health has demonstrated that probiotics can have a strong health-promoting effect [2]. *Bifidobacterium*, a typical human gut bacterium, has been demonstrated to produce surface exopolysaccharide, which can facilitate commensal–host interaction through immune modulation and pathogen protection [7]. In addition, the antitumor activity of probiotics has been well documented in different disease models, which can be attributed to their immunomodulatory or antiproliferation activities [8]. Live biotherapeutic products, a kind of living microbial drugs, have recently attracted extensive attention and been considered as an alternative to traditional prevention and treatment schemes in various clinical environments [9].

However, the properties of probiotics, such as tolerance and colonization in the gut, may vary greatly between species or even strains, which would result in restrictions on their application [10]. Genome engineering of probiotics including the editing of the genome to introduce, remove, or modify phenotypes will improve their tolerance to stress during food production, promote their survival in the gastrointestinal tract, or enhance their probiotic function. The development of genome engineering and synthetic biology has greatly promoted the construction of novel probiotic strains with desired functions, which has facilitated the treatment of metabolic disorders [11], inflammation [12], pathogen infection [13], and even cancer [14,15]. The emergence of various genome editing technologies, including transcription-activator-like effector nucleases, zinc-finger nucleases, CRISPRs, and inducible plasmid self-destruction, assisted genome engineering, has paved the way for the editing of genomes and subsequently improvement in functional modification of probiotics [16,17].

CRISPR-Cas systems are adaptive immune systems of prokaryotes that assist the defense of the host against invading genetic elements such as bacteriophages [18]. Their immune response depends on 3 stages, including adaptation, biogenesis, and interference [19]. At the adaptation stage, the protospacer-adjacent motif (PAM) in the invading DNA is recognized and combined by the adaptation complex, and then the protospacer sequence is inserted into the CRISPR array [20]. A new spacer is also reported to be acquired from RNA via reverse transcription in some CRISPR-Cas systems [21]. At the biogenesis stage, the pre-CRISPR RNA (pre-crRNA) transcribed from the CRISPR array is processed into mature crRNAs by Cas proteins or ribonucleases [22,23]. Each crRNA contains one spacer that matches the target DNA/RNA and part of the repeats. At the interference stage, the ribonucleoproteins of CRISPR-Cas systems bind to and cleave the DNA or RNA target by an RNA-guided DNA or RNA cleavage pattern [24]. With the expansion of microbial resources and the rapid development of bioinformatics, more novel CRISPR-Cas systems and derived CRISPR-Cas variants have been discovered [25]. Thus, the classification of CRISPR-Cas systems has been constantly updated. On the basis of the presence of signature Cas proteins, CRISPR locus structure, and phylogenetic analysis, the latest classification divides CRISPR-Cas systems into 2 classes, 6 types, and 33 subtypes [26]. Class 1 CRISPR-Cas systems, whose effector modules include multiple proteins, consist of type I, III, and IV systems [27]. Class 2 CRISPR-Cas systems occur nearly exclusively in bacteria and perform interference through a single-protein effector complex [28]. Cas9, Cas12, and Cas13 are signature proteins of type II, V, and VI systems, respectively [27].

With the elucidation of the mechanisms for CRISPR-Cas systems, various CRISPR-based technologies have been developed as powerful genome editing tools. Among these technologies, Cas9- and Cas12-based genome engineering have been extensively studied and widely applied because of their high specificity, efficiency, and multiple functions [29,30]. Here, we review the distribution of different types of CRISPR-Cas systems in probiotics, as well as summarize the genome editing strategies based on CRISPR-Cas systems in various probiotics and the application of probiotics engineered by CRISPR-based editing tools in disease treatments. The strategies and challenges for the design of genetically engineered probiotics are also summarized to assist in the construction of desired strains with beneficial effects in the future.

## Diversity of CRISPR-Cas Systems in Probiotics

CRISPR-Cas systems are present in 85.2% of archaea and 42.3% of bacteria [24]. The distribution of this immune system varies greatly among different probiotics that dedicated by Table 1. For the *Lactobacillus* genus, the occurrence rate of complete CRISPR-Cas systems is above 40% [31]. Interestingly, some species contain more than one system in cells, and some other species, such as *Lactobacillus acidophilus*, encode only the CRISPR array and lack associated *cas* genes [32]. The occurrence rate of CRISPR-Cas systems in some *Lactobacillus* species exceeds 90%, such as *Lactobacillus crispatus* (96%) and *Lactobacillus delbrueckii* (93%) [32]. Type II systems, mainly subtype II-A, are the most abundant systems in *Lactobacillus*, whereas type III systems are only found in a few probiotic *Lactobacillus* species, such as *Lactobacillus ruminis* (about 41%) [31]. Besides, 32% of *Lactobacillus salivarius*, recently named *Ligilactobacillus salivarius*, also harbor type III systems [31] . In *Bifidobacterium*, 57% of the strains were detected to harbor CRISPR-Cas systems, including subtypes I-E, I-C, I-G, II-A, and II-C [33]. Among them, the occurrence rate of type I reaches 54%, and type I-E is the major subtype, which is the same as that in *Lactobacillus* [31]. However, type III systems are not found in *Bifidobacterium* [31]. *Streptococcus thermophilus*, whose Cas nucleases have been widely applied in engineering technologies, usually harbors one or more CRISPR-Cas systems, including types I, II, and III [31]. Besides, genome sequence analysis has revealed that only 17% of *Limosilactobacillus reuteri* contains CRISPR-Cas systems [32]; approximately 30% of *Pediococcus acidilactici* strains encode complete subtype II-A CRISPR-Cas systems [34]; and nearly all *Akkermansia muciniphila* harbors subtype I-C CRISPR-Cas systems, while 9% of *A. muciniphila* strains carry both subtype I-C and subtype II-C systems [35]. Moreover, the subtype I-B CRISPR-Cas system has been characterized in *Clostridium butyricum* [36]. CRISPR-Cas systems, mainly type I, have also been commonly found in *Bacillus coagulans* (recently named as *Weizmannia coagulans*), and some strains harbor more than one CRISPR locus in *B. coagulans* cells [37]. However, CRISPR-Cas systems are not found in *Bacillus subtilis*.

**Table 1.** Endogenous CRISPR-Cas systems in probiotics.

**Probiotics**	**Characteristics of CRISPR-Cas systems**	
	**Occurrence in genus**	**Occurrence in species**
*Lactobacillus*	Mainly type II, especially subtype II-A [31]	*L. crispatus*: 96% [32]; types II-A, I-B, and I-E [66]
		*L. delbrueckii*: 93% [32]
		*L. ruminis*: type III (41%) [31]
		*L. acidophilus*: only CRISPR loci and lack of *cas* genes [135]
*L. salivarius*	Type III (32%) [31]	
*L. reuteri*	17% [32]	
*Bifidobacterium*	Occurrence rate: 57%, including subtypes I-E, I-C, I-G, II-A, and II-C [33]; mainly type I (54%), especially type I-E; type II: relatively abundant in *Bifidobacterium longum*; type III: absent [31]	
*B. coagulans*	Type I; some strains harbor more than one system [37]	
*S. thermophilus*	High occurrence (nearly 100%); high abundance (types I-E, II-A, II-C, and III-A); various types can coexist [31]	
*P. acidilactici*	Complete type II-A: 30% [34]	
*A. muciniphila*	Type I-C (majority) and type II-C [35]	
*Bacteroides fragilis*	Types I-B, III-B, and II-C; 32% had 2 types, and 2.7% had both 3 types [136]	
*C. butyricum*	Type I-B [36]	

## CRISPR-Cas System-Based Genome Engineering in Probiotics

With the discovery of CRISPR-Cas systems, more high-efficiency, flexible, and precisely targeted gene editing methods have been established. In general, genetic manipulation based on CRISPR-Cas systems involves generating nucleic acid break at the target site by nuclease, followed by repair to achieve the desired mutation [38]. Cas9 nucleases, which induce DNA double-strand breaks (DSBs) through the histidine–asparagine–histidine and resolvase-like nuclease domain C domains [39,40], have been developed into a common genome engineering tool [41,42]. In recent years, a variety of Cas9 orthologs and variants with a wide range of PAM sequence preferences and even a near-PAMless Cas9 variant (SpRY) have been identified and constructed, which greatly expand the application of Cas9 nucleases [43–46]. Cas12 in type V, cascade-Cas3 complex in type I, and CRISPR-associated complex for antiviral defense subtype M (Csm) or CRISPR-associated complex for antiviral defense subtype R (Cmr) complex in type III are also CRISPR-Cas nucleases that can induce DNA break [47–49]. Currently, various gene editing strategies without dependence on DNA DSBs have been developed. Cas9 nickase (nCas9), a Cas9 variant that only cuts one single strand of DNA, has been widely applied for base editors (BEs) [50] and prime editors [51]. Dead Cas9 (dCas9), whose both nuclease domains are inactivated, can only bind to target DNA but does not generate DNA breaks; it has been used for genetic modification such as CRISPR activation, CRISPR interference (CRISPRi), and epigenetic modification [52,53]. dCpf1 (dead Cpf1)-based BEs have also been successfully developed to perform base editing in A/T-rich regions [54]. Besides, Cas13 nucleases in type VI system and Csm or Cmr complex in type III can cleave and degrade RNA without bias from protospacer flanking sequence [49,55,56].

Repair will be performed by different pathways when nucleic acids in cells are damaged. Nonhomologous end joining (NHEJ) is a major approach for the repair of DNA DSBs in cells without relying on repair templates [57]. NHEJ-based CRISPR tools have been widely used to edit genome, such as gene deletion, insertion, and replacement [58–60]. Notably, it is an error-prone pathway and more common in eukaryotes than in prokaryotes [57]. Microhomology-mediated end joining, which requires microhomologous sequences, is also an error-prone repair approach that can be used for genome manipulation [61]. Gene editing tools based on homology-directed repair (HDR) with repair templates allow more precise introduction of desired changes [41]. The commonly used strategies to optimize HDR-based editing include the suppression of NHEJ activity [62], using of donors with improved stability and Cas nuclease with high activity and the improvement of recombination efficiency [38,63]. As for genetic manipulation of large segments, transposon-associated CRISPR systems are ideal for cells with low HDR efficiencies [64].

### Genome editing using CRISPR-Cas systems in lactic acid bacteria

**Fig. 1.**
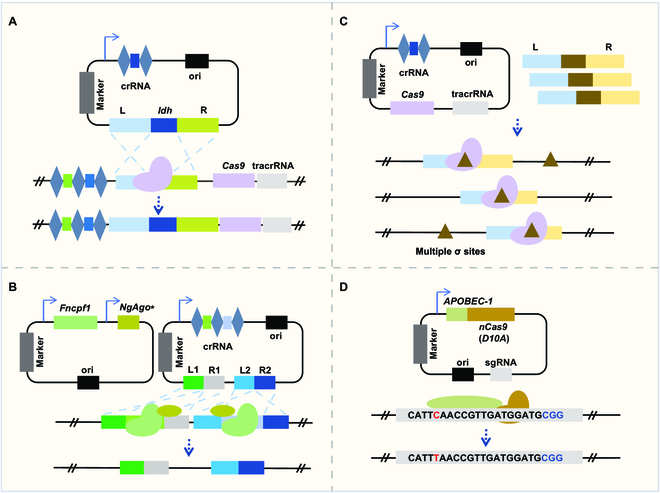
CRISPR-based genome editing in probiotics. (A) Integration of *ldh* gene into chromosome via the endogenous II-A CRISPR-Cas system in *P. acidilactici*. L, left arm; R, right arm; ori, replication origin; crRNA, CRISPR RNA; tracrRNA, trans-activating crRNA; marker, screening marker. (B) CRISPR/Cpf1-assisted multiple genes editing in *B. subtilis*. A mutation of *Natronobacterium gregoryi* Argonaute (NgAgo) was used to improve the homology recombination efficiency. (C) Gene editing based on δ sites of Ty elements in the yeast genome. (D) C·G-to-T·A amber mutation based on the CRISPR-cytosine BE in *Bifidobacterium*. APOBEC-1, a member of activation-induced cytidine deaminase/APOBEC nucleic acid cytosine deaminase family.

Lactic acid bacteria (LAB) are an important group of health-promoting probiotics. The emerging CRISPR-based genome editing technologies make it possible to rapidly identify and genetically modify the functional genes in LAB. Genome editing based on the endogenous CRISPR-Cas systems undoubtedly has obvious advantages; it can avoid the potential cytotoxicity of the exogenous Cas effectors, and the plasmids with smaller sizes are easier to be transformed into cells [65]. Therefore, LAB strains with endogenous CRISPR-Cas systems and the related components in cells can be fully utilized for targeted genome editing. For example, the endogenous subtype I-E CRISPR-Cas system in *L. crispatus* has been applied in flexible and efficient genetic engineering of this strain [66]. Moreover, the endogenous subtype II-A in *P. acidilactici* and subtype II-C in *Lactobacillus gasseri* have been used for enhancing lactic acid production (Fig. 1A) and promoter replacement, respectively [34,67].

Some LAB strains do not encode CRISPR-Cas systems; therefore, heterogeneous systems need to be used for genetic modification and strain improvement. The editing strategies using exogenous CRISPR-Cas systems mainly depend on the following key components: single-guide RNA (sgRNA) for specifically targeting DNA/RNA, Cas effectors for generating DNA/RNA breaks, and repair templates for introducing the desired mutation. Editing tools based on *Streptococcus pyogenes* Cas9 (SpCas9) have been the most widely constructed [68] and successfully applied in LAB strains. Mutations and deletions have been achieved in *Lactibacillus plantarum* using SpCas9 and plasmid-based recombineering [69]. The phage-derived recombinant enzyme RecE/T promotes homologous recombination (HR); hence, the combination of CRISPR-Cas9 with RecE/T is also a powerful strategy for strain engineering [70]. These data have been established in *L. plantarum*, *Lactobacillus brevis*, and *Lactococcus lactis* [70–72]. Besides to the selection of high efficiency, edited cells have been realized in *L. reuteri* by combining CRISPR-Cas9 toolbox and single-stranded DNA recombineering [73]. With the continuous discovery of Cas nuclease variants, the application of CRISPR-based editing has been further developed. The genetic engineering of *Lactobacillus casei* has been improved from plasmid-based HR to a CRISPR-Cas9^D10A^-assisted genome editing system, which allows efficient single-gene deletion or insertion with a shorter cycle time [74]. The engineered CRISPR-nCas9 system has also been successfully developed in *L. acidophilus*, *L. gasseri*, and *Lactobacillus paracasei* [75], highlighting the adaptability of this system in phylogenetically distant *Lactobacillus* species. Besides, CRISPR-dCas9 has already been exploited to perform gene repression in *L. lactis* [76] and functional identification of key cell cycle genes in *L. plantarum* [77].

### Genome editing using CRISPR-Cas systems in *Bacillus*

*B. subtilis* is an excellent industrial starter strain that generally regarded as safe (GRAS) [78]. CRISPR-Cas9-mediated editing technologies have been adopted for *B. subtilis* since 2016 for comprehensive genetic engineering [79–81]. CRISPRi was also achieved in this species using a xylose-induced gene repressor system for higher *N*-acetylglucosamine bioproduction [82]. Later, BEs were developed in *B. subtilis* by utilizing CRISPR-dCas9 and activation-induced cytidine deaminase [83]. In addition, both editing systems assisted by CRISPR-Cpf1 and transcriptional regulation system based on dCpf1 have been constructed in *B. subtilis*, and the synthesis pathways of *N*-acetylglucosamine and acetoin have been engineered in the strain using the Cpf1 system (Fig. 1B) [84].

For some thermophilic *Bacillus* such as *Bacillus licheniformis*, the intracellular activity of exogenous Cas nuclease can be ensured by lowering the cultivation temperature of strains [85]. In *B. licheniformis*, different genome engineering techniques have been achieved, such as single-gene knockout, large DNA fragment deletion, simultaneous disruption of 2 genes, and single-gene integration via the developed CRISPR-Cas9 tools [86]. Moreover, natto kinase activity was found to be markedly increased in *B. licheniformis* recombinant strain DWc9nΔ7 constructed on the basis of the CRISPR-nCas9 system [87]. The CRISPRi system (CRISPR-dCas9) has also been constructed in *B. licheniformis* to improve the production of l-valine [88]. However, because of the low editing efficiency in *B. licheniformis*, it remains very challenging to simultaneously edit multiple genes [87].

### Genome editing using CRISPR-Cas systems in yeast

Some yeast species such as *Saccharomyces cerevisiae* and *Saccharomyces boulardii* are considered probiotics because of their health-promoting functions [89,90]. Owing to its ability to simultaneously edit multiple genes, the CRISPR-Cas system can be used for the reconstruction of complex metabolic pathways in yeast, which was, for the first time, described for *S. cerevisiae* in 2013 [91] In this study, a nearly 100% donor DNA recombination frequency was achieved by cotransformation of a gRNA-expressing plasmid and donor DNA into cells containing a plasmid with constitutive expression of Cas9 [91]. Deletion and mutation were then performed in *S. cerevisiae* using the method described above [92,93]. Various heterologous Cas9-based editing strategies have also been developed for other probiotic yeast species such as *S. boulardii* [94]. Besides editing based on a sgRNA, multiple gene editing has also been realized through the expression of multiple gRNAs in one sgRNA or multiple gRNA cassettes (Fig. 1C) [95]. During this process, Cas9 nuclease can be pretransformed into the cell or integrated into the genome to avoid the subsequent low transformation efficiency. *Francisella novicida* Cpf1 is another powerful tool for yeast genome editing. This nuclease was demonstrated to promote DNA recombination repair with an efficiency up to 100% in *S. cerevisiae* and, thus, facilitate duplex genome editing, deletion of large DNA fragments, and one-step integration of multiple genes [96].

### Genome editing using CRISPR-Cas systems in other probiotics

*Bacteroides* is the most abundant genus in the human gut microbiome and has been linked to a variety of diseases. By assessing the effects of promoters, Cas proteins (SpCas9, SpRY, and FnCas12a), gRNA, and different plasmids on gene editing efficiency, high efficiency, and markerless gene deletion and insertion using the anhydrotetracycline-inducible CRISPR/FnCas12a-based genome editing tool were achieved in multiple human gut *Bacteroides* species [97]. *Escherichia coli* Nissle 1917 (EcN) is well recognized and easy to manipulate, and, therefore, it is commonly used as a therapeutic chassis. In recent studies, the CRISPR-Cas9 system has been applied to remove or engineer native plasmids to enhance the applicability of EcN [98,99]. CRISPR-Cas systems can also be used to modulate specific traits of *Streptococcus* strains to enhance the starter culture phenotype. For example, the endogenous Cas9 nuclease in *S. thermophilus* was reprogrammed to delete the genomic island [100]. Recently, endogenous subtype I-B and heterologous type II CRISPR-Cas9 were used for seamless genome editing in probiotic *C. butyricum* [36]. In *Clostridium tyrobutyricum*, a high butyrate producing bacterium, multiplex genome editing, and high butanol production have been achieved on the basis of its endogenous subtype I-B system [101]. In a more recent work, large DNA fragment knockout and point mutation have been achieved in *Bifidobacterium animalis* subsp. *lactis* by reprogramming the endogenous subtype I-G CRISPR-Cas system and the exogenous CRISPR-cytosine BE [102] (Fig. 1D). *B. coagulans* is an important lactic-acid-producing bacteria, and different CRISPR loci have been identified in its genome [85]. However, there has been no report about genome editing based on CRISPR-Cas systems in *B. coagulans*, which may be attributed to the low transformation efficiency of this species [85].

## Therapeutic Application of CRISPR-Based Engineered Probiotics

Microorganisms in the human body, particularly the gut microbiota, have been demonstrated to affect human health via various ways such as the gut–organ axis [103,104]. The intake of probiotics has been gradually demonstrated as an effective strategy to prevent or mitigate diseases in humans. Among various probiotics, genetically modified probiotic strains have stronger or newer properties and exhibit greater research and application value [105]. Currently, an increasing number of engineered probiotics, which are mostly constructed by traditional plasmid expression or HR, are applied to the prevention or treatment of various diseases [106]. For example, lambda red recombineering was used to carry out gene knockout and gene integration in EcN, and the engineered strain could transform ammonia, a metabolic product of tumor cells, into l-arginine, thereby increasing the number of tumor-infiltrating T cells and playing an antitumor role [107]. Engineered *L. reuteri*, which expresses the anti-inflammatory cytokine interleukin-22 through plasmid, can promote the expression of antimicrobial C-type lectin regenerating islet-derived 3 gamma in the gut, thereby alleviating alcohol-induced liver disease [108].

With the rapid development of CRISPR-based editing technology and synthetic biology, probiotics engineered on the basis of CRISPR-Cas systems have been gradually developed for microbial drugs. As is known to all, the emergence of antibiotic-resistant bacteria has become a serious global threat [109]. Currently, various strategies based on CRISPR-Cas technologies have been utilized to study the mechanism of antibiotic resistance and treat infectious diseases. For example, a high-efficiency conjugative delivery vehicle for CRISPR-Cas9 has been generated for antimicrobial therapy. The engineered conjugative probiotic EcN was found to eliminate nearly all targeted antibiotic-resistant *E. coli* strains from the gut microbiota (Table 2) [110]. This study contributes to important technological innovation for the use of CRISPR-Cas systems to deal with antibiotic-resistant bacteria. Besides, EcN strain vaccine with defense against F4^+^/F18^+^ enterotoxigenic *E. coli* (ETEC) infection was developed by integrating F4 and F18 fimbriae cluster genes into the chromosome through the CRISPR-Cas9 system (Table 2) [111]. The results demonstrated that serum antibodies from the immunized mice and piglets could significantly inhibit the adherence of F4^+^ and/or F18^+^ ETEC strains to porcine intestinal cell lines in vitro. Inflammatory bowel disease is a complex chronic inflammatory disorder of the gastrointestinal tract. The self-tunable engineered *S. cerevisiae* strain BS016 with the expression of a human P2Y2 purinergic receptor and the secretion of the adenosine 5′-triphosphate-degrading enzyme apyrase was constructed based on the CRISPR-Cas9 system (Table 2) [12]. This engineered yeast can sense proinflammatory molecules and generate a proportional self-regulated response, thereby inhibiting intestinal inflammation in mouse models of inflammatory bowel disease. Recently, the engineered butyrate-producing *B. subtilis* strain BsS-RS06551 was constructed on the basis of the CRISPR-Cas9 genome editing system, which could exert positive intervention effects on obesity and metabolic regulation in mice fed with a high-fat diet (Table 2) [112]. Evidently, engineered probiotics have great potential for relieving various diseases as live biotherapeutic products.

**Table 2.** Therapeutic applications of engineered probiotics based on CRISPR-Cas systems.

**Diseases**	**Applications**
Infectious diseases	The engineered EcN delivers CRISPR-Cas9 in gut and thus eliminates antibiotic-resistant bacteria [110].
	The engineered EcN integrated with F4 and F18 fimbriae cluster genes using CRISPR-Cas9 technology could defend against the infection of F4^+^/F18^+^ ETEC [111].
Inflammatory bowel disease	The engineered yeast strain BS016 expressing human P2Y2 purinergic receptor and secreting adenosine 5′-triphosphate-degrading enzyme apyrase through CRISPR-Cas9 can sense and then degrade a proinflammatory molecule [12].
Obesity	The engineered *B. subtilis* strain BsS-RS06551 edited by CRISPR-Cas9 system produces butyrate and regulates metabolic to prevent obesity [112].

## Strategies and Challenges for CRISPR-Based Genome Editing of Probiotics in the Future

**Fig. 2.**
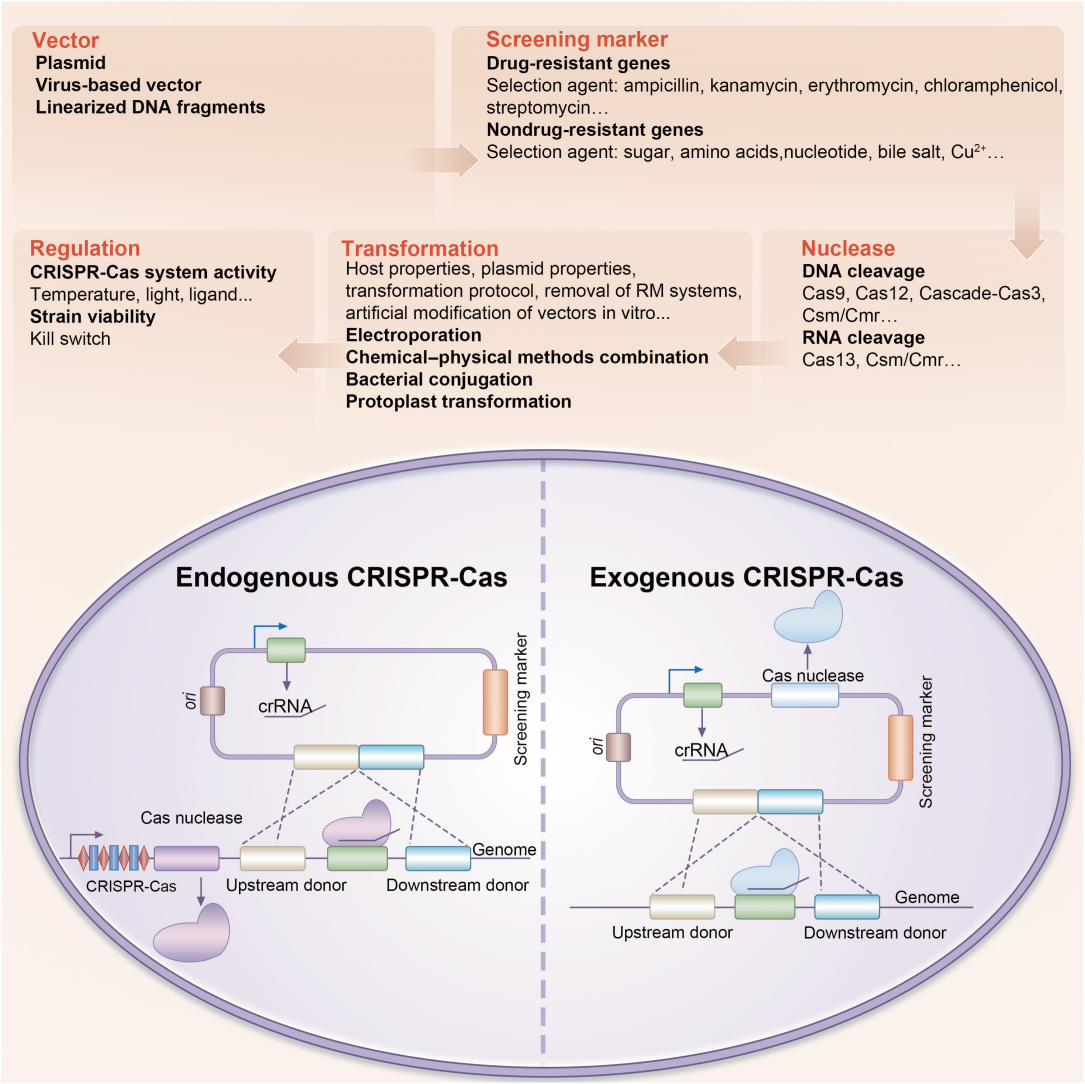
CRISPR-based genome editing strategies for probiotics. Top portion: Key factors that should be fully considered in CRISPR-based genome editing. Bottom portion: Schematic of genome editing based on endogenous CRISPR-Cas system (left); schematic of genome editing based on exogenous CRISPR-Cas system (right). RM, restriction–modification.

Obviously, the premise for genome manipulation of probiotics is the delivery of an editing toolbox into cells regardless of the editing method. Electroporation is a common method for the transformation of probiotics such as LAB [113]. To improve the transformation efficiency, the following controllable factors should be fully considered and continuously optimized: including host properties (cell wall composition, growth stage, final cell density, and the presence of endogenous plasmid), plasmid properties (plasmid source, plasmid concentration, and replication origin), and transformation conditions (electrical field strength, electric resistance, pulse duration, buffer, resuspending culture media, and recovery time) (Fig. 2) [17]. Besides, artificial modification of vectors in vitro [114], removal of restriction–modification systems in cells [115], and multiple repetitions of transformation and plasmid elimination can help the vectors evade host defense [116], so as to improve the electroporation efficiency. Besides electroporation, some other technologies, such as physicochemical methods [117], bacterial conjugation [118], protoplast transformation [119], and natural competence [120] are all candidate approaches for vector delivery.

Proper screening after transformation is essential for the selection of desired strains. It is known that screening based on antibiotic resistance is the most frequently used method for obtaining engineered strains [121]. Common resistance genes against ampicillin, kanamycin, chloramphenicol, erythromycin, zeocin, or tetracycline should be selected according to the antibiotic sensitivity of the host (Fig. 2 and Table 3). Considering the high cost of antibiotics for eukaryotes and the risk of horizontal transfer of resistance genes, auxotrophic marker genes related to sugar, amino acid, and nucleotide metabolic pathways can also be used as genetic screening markers (Fig. 2) [122]. Moreover, glucosamine synthase gene and stress-resistant protein-encoding genes such as *bsh*, which encodes bile hydrolase that have been successfully applied as screening markers [123,124], can also be considered.

**Table 3.** Plasmids used for genome editing in probiotics.

**Probiotics**	**Plasmids**	**Characteristics**	**Addgene**
*L. crispatus*	pTRKH2 [66]	High copy, p15A ori, Erm^r^	#71312
*P. acidilactici*	pMG36e [34]	High copy, pWV01 ori, Erm^r^	#133853
*L. gasseri*	pGK12 [67]	pMB1 ori, Amp^r^	#186342
*Lactobacillus plantarum*	pSIP403 [77]	pBR322 ori, Erm^r^	#122028
*L. reuteri*	pVPL3004 [73]	Cat^r^	
*L. casei*	pIB184 [74]	Low copy, pBR322 ori, Erm^r^	#90194
*Lactobacillus lactis*	pHSP02 [137]	Low copy, pSC101 ori, Kan^r^	#117259
*B. subtilis*	pHT01 [80]	pBR322 ori, Amp^r^, Cat^r^	
	pCas9 [79]	Low copy, pBR322 ori, Cat^r^	#167547
	pLCx [82]	Cat^r^	
*B. licheniformis*	pWH1520 [86]	pBR322 ori, Amp^r^, Tet^r^	
	pHY300PLK [87]	Amp^r^, Tet^r^	
*W. coagulans*	pMSR10 [138]	ColE1 ori, Amp^r^, Cat^r^	
	pNW33N [138]	pBR322 ori, Cat^r^	
*S. cerevisiae*	Cas9-NAT [92]	pBR322 ori, Amp^r^, Nrs^r^	#64329
	p415 Gal-Lp/p414 TEF1p [91]	Low copy, Amp^r^	
	pRS415, pRS42H [139]	Amp^r^	
	pCas [12]	Low copy, pBR322 ori, Kan^r^	#60847
*S. boulardii*	pRS42H [94]	pBR322 ori, Amp^r^	#177155
EcN	pCas9-KT [98]	ation ori, Kan^r^	
*S. thermophilus*	pOR128 [100]	Low copy, Erm^r^	
*C. butyricum*	pJZ23-Cas9 [36,101]	pBP1 ori, ColE1 ori, Amp^r^, Cat^r^	

Considering the toxicity of heterogeneous Cas nucleases [125], gene editing tools established on the basis of endogenous CRISPR-Cas systems in cells should be a priority for genome engineering. For probiotics harboring endogenous CRISPR-Cas systems, functional validation of the system such as interference activity detection and PAM sequence identification should be conducted first. Considering the standardization of construction and stability of the engineered probiotics, synthetic biology modules such as vector, origin, promoter, and signal peptide should all be designed and optimized (Fig. 2 and Table 3). To reduce the off-target events induced by DNA cleavage during genome editing, new nucleases such as dCas9 and dCpf1, which do not introduce DNA double-strand cleavage, have been developed to ensure the accuracy of genome editing [126,127].

Although CRISPR-based genome editing has made great progress, there are still some public concerns about engineered probiotics. Microbial biocontainment is one of the greatest challenges. Technologies such as kill switches can be applied to construct probiotic chassis with controllable viability (Fig. 2) [128]. Another obstacle to the clinical application of genetically modified probiotics is safety, such as the immunogenicity of functional proteins involved in gene editing and the potential for inflammation caused by preexisting antibodies against CRISPR components [129]. More importantly, regulating the activity of the CRISPR-Cas system for genetic manipulation is undoubtedly an ideal strategy to ensure the safety of clinical treatment with engineered probiotics. Temperature [130], light [130,131], and ligands [132–134] can all be used to regulate the activity of the editing systems (Fig. 2). Moreover, it is important to determine how to ensure the stability of engineered probiotics, how to clarify their mechanism of action, and how to promote their application in the market. Undoubtedly, the use of engineered probiotics to promote the development of animal and human health industries will face great opportunities and challenges in the future.
